# Clinical management and microscopic characterisation of fatique-induced failure of a dental implant. Case report

**DOI:** 10.1186/1746-160X-2-18

**Published:** 2006-06-22

**Authors:** S Capodiferro, G Favia, M Scivetti, G De Frenza, R Grassi

**Affiliations:** 1 Department of Dental Sciences and Surgery, University of Bari-Italy, Piazza G. Cesare, 11,70124 Bari –, Italy

## Abstract

**Background:**

Osseointegrated endosseous implants are widely used for the rehabilitation of completely and partially edentulous patients, being the final prosthodontic treatment more predictable and the failures extremely infrequent. A case of fracture of an endosseous dental implant, replacing the maxillary first molar, occurring in a middle-age woman, 5 years after placement is reported.

**Materials and methods:**

The difficult management of this rare complication of implant dentistry together with the following rehabilitation is described. Additionally, the authors performed an accurate analysis of the removed fractured implant both by the stereomicroscope and by the confocal laser scanning microscope.

**Results and discussion:**

The fractured impant showed the typical signs of a fatigue-induced fracture in the coronal portion of the implant together with numerous micro-fractures in the apical one. Three dimensional imaging performed by confocal laser scanning microscope led easily to a diagnosis of "fatigue fracture" of the implant. The biomechanical mechanism of implant fractures when overstress of the implant components due to bending overload is discussed.

**Conclusion:**

When a fatigue-induced fracture of an dental implant occurs in presence of bending overload, the whole implant suffers a deformation that is confirmed by the alterations (micro-fractures) of the implant observable also in the osseointegrated portion that is easily appraisable by the use of stereomicroscope and confocal laser scanning microscope without preparation of the sample.

## Introduction

Osseointegrated endosseous implants are widely used for the rehabilitation of completely and partially edentulous patients, being the final prosthodontic treatment more predictable and the failures extremely infrequent [1-3]. Nevertheless, in the *iter *of realization of an implant retained prosthesis a series of possible complications have to be considered by the implant/prosthetic team: these are usually distinguished in surgical (disturbance, parae/anaesthesia, haematoma, mandibular fractures, haemorrhage, tooth necrosis) [4,5] that are the most frequent ones, and mechanical (screw loosening, screw fractures, framework fractures, veneering resin or ceramic fractures, problems of mechanical retention) [6,7]. Instead, implant fracture is very infrequent but, when it occurs, its management is very challenging because of its surgical, rehabilitative and emotional implications, and often also for insurance reasons [8].

The aim of this report is to describe the pathology and the management of a case of implant fracture occurring in the posterior maxilla of a middle age woman who was rehabilitated by implant-retained partial fixed prosthesis; an accurate microscopic evaluation by using stereo and confocal laser scanning microscopy of the fractured implant and the surrounding bone was performed demonstrating that it was a "fatigue fracture" of the implant due to increased stress and bending overload forces originates from inadequate implant diameter in relation to the site needing rehabilitation.

### Case report

**Figure 1:**
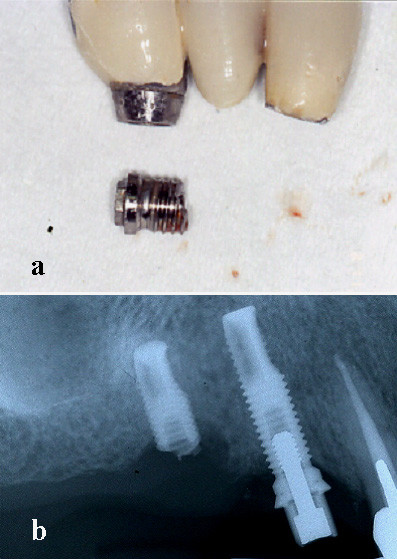
The removed metal-ceramic prosthesis with the fractured implant (a); radiological appearance of the fractured implant replacing the first molar (b).

**Figure 2:**
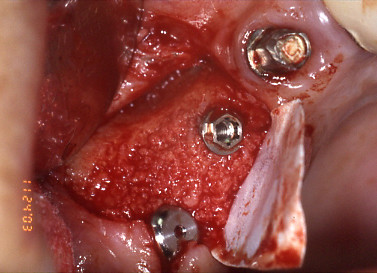
Three months later, two implants were placed in the regenerated bone.

A middle-age woman was referred to our department for raised mobility of a fixed implant-supported prosthesis replacing the first and second right maxillary premolar and the first molar; the patient referred that implants were placed in September 1996 and loaded after six months. The clinical examination revealed a cemented metal-ceramic prosthesis supported by two thread osseointegrated implants which was easily removed together with the fractured coronal portion; radiographic examination showed the residual portion of the implant (external hex RP Brånemark implant) in 1.6 position and a marginal crater-like bone loss; no signs of perimplantitis or lack of osseointegration were observable (Fig. 1a, b). Under local anaesthesia a mucoperiostal flap was elevated exposing the fractured implant which was removed by a trephine cutter and the surgical site was regenerated with calcium sulphate (Surgiplaster) also to promote ridge augmentation. Three months later, two thread implants of length and diameter adequate to crestal bone dimension (Brånemark, Nobel Biocare, MKIII, 5 × 10 mm in 1.6 position and 3,75 × 13 mm in 1.5 position), were placed in the regenerated bone (Fig. 2); four months later, a cemented gold-ceramic prosthesis was positioned upon the prosthetic abutments.

**Figure 3:**
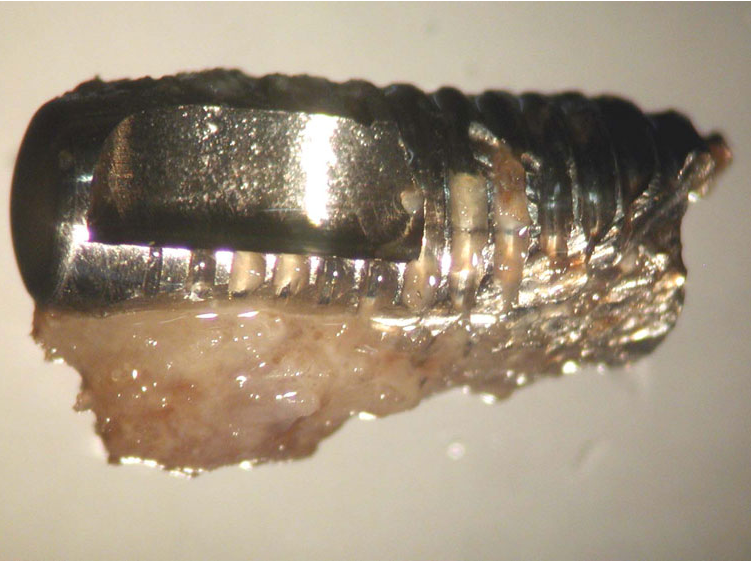
Stereomicroscope imaging showing well-osseointergated fractured implant.

**Figure 4:**
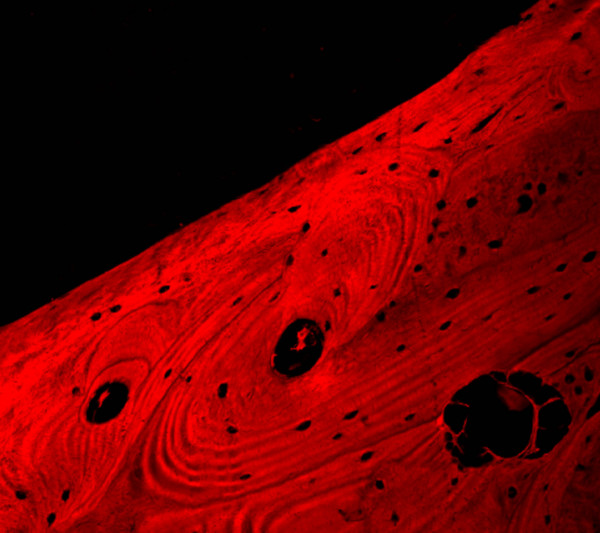
Confocal laser scanning microscopic analysis of the perimpant bone showing no sign of perimplantitis or fibrous integration of the fractured implant; no inflammatory infiltrate is observable at the interface bone-implant.

The fractured implant was observed both at the stereo-microscope and at the confocal laser scanning microscope; it totally measured 3,75 mm of diameter and 13 mm of length and was transversally fractured at the level of the third-fourth thread; the endosseous residual portion was well-osseointegrated as confirmed by the histological evaluation of the perimplant bone which showed no signs of perimplantitis or fibrous integration of the implant (Fig. 3 and 4).

**Figure 5:**
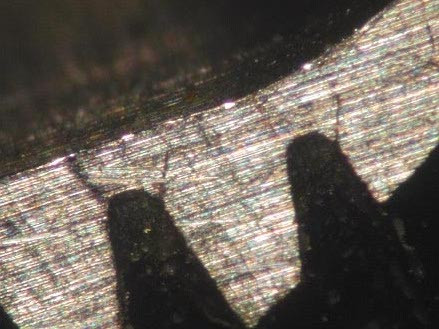
Stereomicroscope analysis: particular of apical screws showing well-defined initial fracture rims at the basis of the screws.

After removed the bone, at the stereomicroscope the fracture surface presented the typical features of the fatigue fracture observed by other authors using fractography with scanning electronic microscope (main transversal fracture surface with a variable number of fatigue striations indicating the advancement of the crack front under cycling loading)[11-13]; besides, numerous surface irregularities were also detected on the roots of the screw thread of the well-osseointegrated apical portion of the implant (Fig. 5), probably related to microfractures or cracks, defects of industrial workmanship or finishing touch, or alterations due to implant removal by burs.

**Figure 6:**
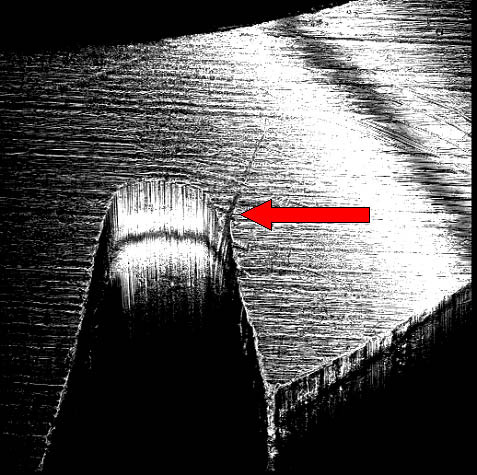
3D CLSM reconstruction: absence of any other damage of the implant surface can exclude other causes than fatigue fracture.

**Figure 7:**
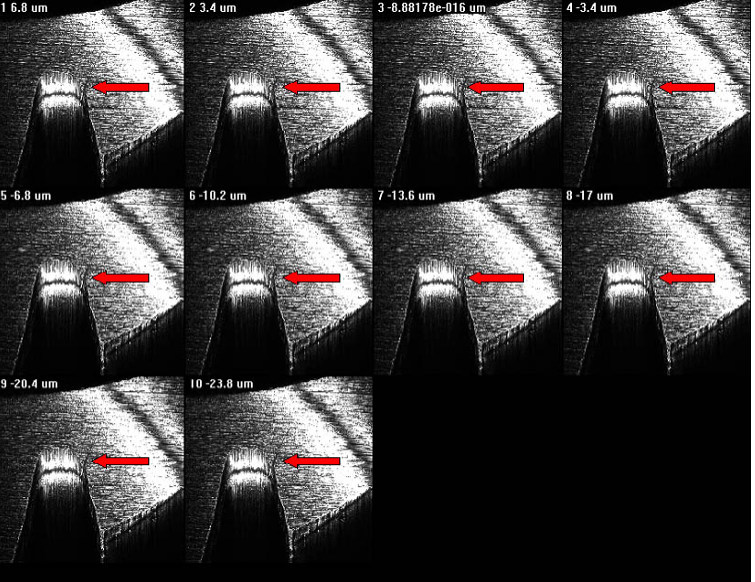
3D CLSM reconstruction: the serial plan scanning imaging shows the complete micro-fracture of the apical screw.

For such distinction authors used confocal laser scanning microscope (Nikon Eclipse C1) with the following configuration: surface scanning method by using Argon-ion laser source at 488 nm wavelength; 1.92 msec time of scanning, B/W imaging, 10 steps of 3D scanning with plans at 3.4 μm distance, volume rendering 3D rewconstruction mode. The confocal laser scanning microscopy (CLSM) allowed to perform an analysis of the surface both with three-dimensional reconstruction and with an evaluation of the depth of the defects by serial scanning at different focal plans (10 different plans at 3,4 μm of distance) as showed in figure 6 and 7.

Considering the appearance on 3D reconstruction and the presence of the defects in all scanned plans, authors agreed in defining them as microfractures or craks of the apical portion of the implant though well-osseointegrated due to fatigue.

## Discussion

Excluding lack of osseointegration and perimplantitis, the main causes of dental implant failure can result from technical problems relating both to prosthesis and to implant components. The latter includes above all abutment screw fracture, which is a complication with an increasing rate of incidence, and implant fracture which remains extremely rare [9-12]. These were first described by Brånemark et al. in 1977, who reported on 13 fractured implants on a total of 1618 osseointegrated implants. Since then, only sporadic cases or short cases series are been reported; in fact, reviewing the international literature till to 2002 [1,2,5-8,10,14,20], only 238 cases of implant fractures have been reported on a total of almost 24000 implants placed with a extremely variable percentage value of incidence, ranging from 0 to 7,2%. Nevertheless, almost all the authors agree in sustaining that the *"bending overload"*, a term introduced by Rangert et al. in 1995 [13], is the main prosthesis-related cause leading to fatigue fracture of implants [14-17].

The mechanism of fracture has a multi-factorial aetiology; when number, position, dimension, design of implant and restoration are inadequate to the site needing rehabilitation, the situation of bending overload is present and an initial bone loss around the implant begins. If no correction of the prosthesis is introduced, the coronal screws become as soon as exposed and a crater-like appearance of the surrounding bone is observable. At this time, the coronal portion of the implant represents a *lucus minoris resistenziae*, being the implant internally filleted and consequently extremely thin, upon which overstress promotes the creation of numerous micro-fractures that can result, after a variable time range, in a complete fatigue fracture. Ideally, implants with internal abutment connection should be more suitable to this complication.

Besides, the findings of cracks on the root of the screw thread led us to sustain that bending overload creates a deformation of whole implant with consecutive micro-fractures, as previously described in experimental studies [18-21]. The observation of the cracks we performed at the confocal laser scanning microscope, is surely more easily executable unlike other techniques for metal fracture analysis, as no preparation of the sample is necessary; in fact, it leads rapidly to a diagnosis of fatigue fracture by the 3D reconstruction and the examination on several confocal plans of the cracks of the root of the screw thread; in this way defects or alterations of implant surface due to industrial workmanship or surface treatment to promote and increased osseointegration can be easily excluded. Additionally, this investigations could be useful for insurance reasons when failure of a dental implant occurs, while they are surely helpful in the planning of the new implant rehabilitation.

## Competing interests

The author(s) declare that they have no competing interests.

## Authors' contributions

All authors have contributed equally in the realization of this paper.
